# Ergot in Purpura Hemorrhagia

**Published:** 1876-03

**Authors:** J. B. Richardson


ERGOT IN PURPURA HEMORRHAGIA—*By J. B. Richardson, M.D.* On the 2d day of August, 1875, I took charge of the following case: A lad, eleven years of age, who had been delicate for the previous five years, but the subject of no known disease, and generally active and sprightly, had exhibited some lassitude for a week. He had been somewhat exposed from bathing in a creek during a visit to the country ten days before. Upon careful examination I found temperature in axilla to be 102; pulse 110; appetite capricious; palpitation of heart and some fugitive pains in chest and joints; the tongue was slightly furred, and two or three hæmic spots were found on its surface, which were tender and bled upon being touched. I also found hæmic spots on legs, thighs, and body, some of which were larger than a silver dollar. The patient had been chilly the evening before. I ordered fifteen grains of quinine in five doses, repeated at intervals of three hours. As the bowels were somewhat costive, I also gave him a mild aperient of calomel and comp. ext. of colocynth.

The next day, August 3d, I found the purgative medicine to have acted efficiently, and the quinine exhibited no special effect. Fresh hæmic spots were discovered, while those observed on yesterday had become paler. I now gave him a mixture containing gallic acid, aromatic sulphuric acid, and tincture of cinnamon, in water, in free doses every two or three hours. Lemonade and acid fruits were allowed *ad libitum.*

August 4th—Found several new hæmic spots on shoulders and chest. Treatment continued.

August 5th—The hæmic spots in various parts of the body had increased in number and size. Finding the patient more prostrate, and the hæmic spots so much increased that I feared the advent of hemorrhage, I determined to give him ergot, with a view of forestalling this result. I gave twenty drops of the fluid extract, repeated every four hours.

August 6th—Fewer new spots. Ergot continued.

August 7th—No new spots.

August 8th—No new spots. Ergot continued; dose at intervals of eight hours, and to this treatment I added one grain and a half of iron by hydrogen three times daily. During the period I was giving the ergot the pulse gradually diminished in frequency from 110 to the minute to 90, the temperature of axilla also falling from 102 to the normal standard.

August 9th—Continued ergot and iron. Patient manifestly better, with appetite for food; pulse 90; temperature normal; hæmic spots fading and no new ones appearing.

August 10th—the patient still improving. I ordered the ergot and iron to be discontinued. After this date I directed small doses of cod-liver-oil emulsion, also comp. tinct. of cinchona with nitro-muriatic acid.

On the 17th of August patient appeared to be in his usual health. The remains of purpuric spots were but faintly visible,

January 25, 1876—Family report health good.

The underlying conditions which give rise to purpura are not known, and I cannot of course say that they were modified by the use of ergot. Certainly, however, within thirty-six to forty-eight hours after its exhibition no fresh hæmic spots appeared, while under the use of gallic acid, etc., there was no apparent improvement.

I was induced to try the remedy from a knowledge of its physiological action upon the blood-vessels and its usefulness in various hemorrhages. My first object in giving it was to prevent threatened hemorrhage. Since I treated the case I find ergot mentioned by Flint in his practice as a remedy in purpura used by Henock, of Germany. As purpura is a grave disease, I have thought any testimony in favor of a remedial agent worthy of publication. Certainly, if another case should come under my charge, I should give ergot with some confidence, but not to the neglect of other remedies which experience has proved useful.—*Louisville Medical News.*

Calhoun, Ky., February, 1876.

